# Estimating the preclinical Alzheimer's disease course with multimodal data

**DOI:** 10.1002/alz.70658

**Published:** 2025-09-03

**Authors:** Diana L. Townsend, Michael J. Properzi, Tobey J. Betthauser, Hannah M. Klinger, Rory Boyle, Gillian Coughlan, Bernard J. Hanseeuw, Hyun‐Sik Yang, Karly A. Cody, Rebecca E. Amariglio, Michelle Farrell, Heidi I. L. Jacobs, Zahra Shirzadi, Wai‐Ying Yau, Julie C. Price, Jasmeer Chhatwal, Dorene M. Rentz, Keith A. Johnson, Reisa A. Sperling, Aaron P. Schultz, Rachel F. Buckley

**Affiliations:** ^1^ Department of Neurology Massachusetts General Hospital, Harvard Medical School Boston Massachusetts USA; ^2^ Department of Medicine University of Wisconsin–Madison School of Medicine and Public Health Madison Wisconsin USA; ^3^ Wisconsin Alzheimer's Disease Research Center University of Wisconsin School of Medicine and Public Health Madison Wisconsin USA; ^4^ Penn Frontotemporal Degeneration Center Department of Neurology University of Pennsylvania Philadelphia Pennsylvania USA; ^5^ Department of Radiology Massachusetts General Hospital, Harvard Medical School Boston Massachusetts USA; ^6^ Department of Neurology Cliniques Universitaires Saint‐Luc, Institute of Neurosciences, UCLouvain Brussels Belgium; ^7^ Neurology and Neurological Sciences, Stanford University Palo Alto California USA; ^8^ Department of Neurology Center for Alzheimer Research and Treatment Brigham and Women's Hospital, Harvard Medical School Boston Massachusetts USA; ^9^ Athinoula A. Martinos Center for Biomedical Imaging Department of Radiology Massachusetts General Hospital Charlestown Massachusetts USA; ^10^ Melbourne School of Psychological Sciences University of Melbourne Melbourne Victoria Australia

**Keywords:** Alzheimer's disease, amyloid accumulation, cognitive decline, disease time

## Abstract

**INTRODUCTION:**

In observational studies of preclinical AD, an arbitrary “baseline” can obscure where an individual is located along a theoretical continuum. Optimizing longitudinal trajectories can distill multiple, non‐linearly distributed observations into a single metric and inform where an individual may be along the disease course.

**METHODS:**

We developed a cognitive time (c‐time) metric based on longitudinal cognitive data (mean = 7.4 years, range = 1.7–11.6) from 316 participants from the Harvard Aging Brain Study using non‐linear least‐squares optimization. We examined path analyses between a published time‐to‐amyloid beta (Aβ)+ metric (longitudinal Aβ positron emission tomography [PET]: mean = 6.3 years, range = 4.2–9.7) and c‐time, including demographics, brain volume, cortical thickness, tau PET, and cardiovascular risk.

**RESULTS:**

Time‐to‐Aβ+ and c‐time were positively correlated, with time‐to‐Aβ+ possessing direct and indirect associations with c‐time through regional tau PET, hippocampal volume, and cortical thickness.

**DISCUSSION:**

Optimizing longitudinal multimodal data to estimate a theoretical continuum can provide unique and age‐independent information about the distance an individual might be from disease‐related events.

**Highlights:**

- Cognitive time (c‐time) represents an individual's distance from a hinge point of cognitive decline.
- C‐time and time‐to‐amyloid beta (Aβ)+ were moderately positively correlated.
- Inferior temporal tau and cortical thickness mediated the effect between c‐time and time‐to‐Aβ+.
- C‐time more closely associates with tau, brain atrophy, and cortical thickness.

## 1 BACKGROUND

The progression of Alzheimer's disease (AD) is characterized by an insidious accumulation of amyloid and tau, followed by cognitive decline, occurring over many years.^1^, ^2^ Defining the position of a single individual along the AD clinicopathological cascade in the sporadic case, however, is challenging. Unlike autosomal dominant AD (ADAD), pathological and clinical trajectories are highly heterogeneous.^1^ ADAD provides an interesting example in which an estimated time to onset of a clinical diagnosis can be closely anchored to parental age of onset to dementia.^3^ Models attempting to estimate underlying disease time for sporadic AD have so far been based on limited follow‐up, and with variability in patient age and progression rate. Further limitations are that data can often be left censored, meaning few individuals have progressed to dementia, and obscuring a “time to event” to anchor a disease time metric. Leveraging more than a decade of clinical and neuroimaging follow‐up across hundreds of clinically normal older adults (at baseline) from the Harvard Aging Brain Study (HABS), our goal was to build a multimodal time scale of progression to AD to provide unique insights into the natural history of the disease.

Optimizing and integrating information from longitudinal cognitive or biomarker trajectories can distill multiple, heterogeneous, and non‐linear observations into a single metric, allowing for the estimation of an individual's location along the AD continuum. Some theoretical frameworks have been proposed that portray the progression of the disease as a time scale of AD. Multiple models have estimated time to amyloid beta (Aβ) onset based on trajectories of amyloid accumulation,^4^, ^5^, ^6^ implementing methods such as linear mixed effects models, logistic growth models, and group‐based trajectory modeling.^4^, ^6^, ^7^ Most recently, Betthauser et al. developed the sampled iterative local approximation method (SILA), which robustly aligns multiple short‐term amyloid trajectories into one long‐term continuum.^5^ Others have postulated disease time models using linear mixed effects models on cognition^8^ or a combination of biological measures and cognition to develop a disease time scale.^9^, ^10^ Few studies have compared disease time metrics across mixed domains, such as Aβ positron emission tomography (PET) and cognitive assessment.^9^, ^11^

Our goal in this paper was to optimize longitudinal and non‐linear cognitive trajectories from the HABS into a single metric that represents the full time scale of cognitive decline (referred to as c‐time). We further applied Betthauser et al.’s SILA method to determine the time‐to‐Aβ+. Integrating these two metrics, we then examined direct and indirect associations between c‐time and time‐to‐Aβ+ in conjunction with demographics, cardiovascular risk, and neuroimaging biomarkers of tau burden and neurodegeneration.

## 2 METHODS

### 2.1 Standard protocol approvals, registrations, and patient consents

Data for these analyses came from the HABS. Procedures for HABS were approved by the Massachusetts General Brigham (MGB) Human Research Committee, the institutional review board for MGB hospitals (Protocol: 2010P000297). All participants gave written informed consent.

### 2.2 Participants and data

**FIGURE 1:**
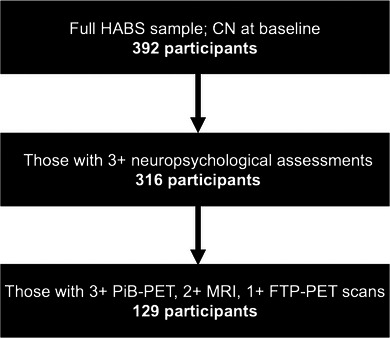
Diagrammatic flow of the number of HABS participants included for each analysis. The 129 participants were used for each of our models, including the creation of c‐time. CN, cognitively normal; c‐time, cognitive time; FTP, flortaucipir; HABS, Harvard Aging Brain Study; MRI, magnetic resonance imaging; PET, positron emission tomography; PiB, Pittsburgh compound B

For optimization of cognition and Aβ, we selected two different samples of clinically normal older adults from HABS to optimize our time‐scaling models within each modality. See Figure 1 for the diagrammatic flow of participant selection. For the first sample, we selected participants with three or more neuropsychological assessments (*n* = 316, mean = 7.4 years, range = 1.7–11.6 years, age = 71.5 [± 8] years, 60% female). This sample was used exclusively for the optimization of cognition. For the second sample, which was used for the optimization of Aβ and subsequent analyses, we selected participants with at least three ^11^C‐Pittsburgh compound B (PiB) PET scans (*n* = 129, mean = 6.3 years, range = 4.2–9.7 years, age = 72.8 [± 5.9] years, 62% female; see Table 1 for demographics). Participants in both samples were required to have at least two structural magnetic resonance imaging (MRI) scans available for longitudinal analysis. Given that ^18^F‐flortaucipir (FTP) PET was introduced mid‐way through the study, we selected participants based on the availability of at least one FTP PET scan. Baseline neuropsychological assessments were matched to the closest first PET scan, with a mean difference of 0.41 years (± 0.28) for PiB PET and 0.28 years (± 0.17) for tau PET.

**TABLE 1:** Baseline demographics stratified by Aβ status.

Characteristic	Aβ–^a^, *N* = 94^b^	Aβ+^a^, *N* = 35^b^	*p* value^c^
**Sex**			0.2
Female	61 (64.89%)	18 (51.43%)	
Male	33 (35.11%)	17 (48.57%)	
**Age**	72.16 (6.11)	74.60 (4.99)	**0.009**
**Race**			0.7
White	84 (89.36%)	30 (85.71%)	
Black	9 (9.57%)	5 (14.29%)	
Native American	1 (1.06%)	0 (0.00%)	
**Ethnicity**			>0.9
Non‐Hispanic	93 (98.94%)	35 (100.00%)	
Hispanic	1 (1.06%)	0 (0.00%)	
**Education**	16.28 (3.03)	16.86 (2.70)	0.4
***APOE* ε4**			**<0.001**
ε4–	79 (84.04%)	14 (40.00%)	
ε4+	15 (15.96%)	21 (60.00%)	
**Entorhinal cortex FTP SUVR**	1.09 (0.09)	1.21 (0.16)	**<0.001**
**Inferior temporal FTP SUVR**	1.18 (0.06)	1.28 (0.15)	**<0.001**
**Hippocampal volume adjusted**	7521.43 (711.66)	7180.77 (593.92)	**0.009**
**Gray matter volume adjusted**	577,370.29 (26,201.65)	570,529.17 (28,904.53)	0.075
**Cortical thickness**	2.98 (0.10)	2.89 (0.15)	**<0.001**
**Cardiovascular disease risk**	28.62 (17.16)	37.58 (17.49)	**0.008**

^a^ Cut‐off for amyloid positivity with the longitudinal pipeline is 0.87 DVR.

^b^ n (%); mean (SD).

^c^ Pearson chi‐squared test; Wilcoxon rank sum test; Fisher exact test.

Abbreviations: Aβ, amyloid beta; *APOE*, apolipoprotein E; DVR, distribution volume ratio; FTP, flortaucipir; SD, standard deviation; SUVR, standardized uptake value ratio.

### 2.3 Cognitive composite

The Preclinical Alzheimer Cognitive Composite‐5 (PACC‐5) has been previously reported.^12^ In brief, baseline‐anchored *z* scores are computed and averaged across the following tests: the Mini‐Mental State Examination, Logical Memory Delayed Recall, Digit‐Symbol Substitution Test, Free and Cued Selective Reminding Test (both cued and free recall), and Category Fluency.

### 2.4 Aβ and tau PET neuroimaging

**RESEARCH IN CONTEXT**

1. **Systematic review**: The authors examined the literature on disease time models for Alzheimer's disease (AD). Current literature details heterogeneous data in the age of onset and progression rate, limited follow‐up, and non‐linear trajectories.
2. **Interpretation**: Longitudinal data across amyloid and cognition modalities can place individuals on continuums of amyloid and cognitive disease time, respectively. Time‐to‐amyloid beta (Aβ)+ was moderately correlated with cognitive time (c‐time), with direct and indirect statistical pathways between the two via tau burden, hippocampal volume, and cortical thickness.
3. **Future directions**: Next steps are to expand this approach to other modalities (i.e., tau positron emission tomography, plasma) and larger datasets comprising pooled cohorts, which may allow for more robust models of the natural history of preclinical AD.

Neocortical Aβ burden was acquired using PiB PET. Acquisition parameters and details of the longitudinal pipeline are published elsewhere.^13^ We used a distribution volume ratio (DVR) gathered from 60 minute time series and calculated using the Logan method.^14^ The target region was a global composite of neocortical regions representing frontal, lateral, and retrosplenial (FLR) regions, and a reference region optimized for longitudinal Aβ PET was created from a composite of cerebellar gray and eroded white matter.^15^ The threshold for Aβ positivity of 0.87 DVR was determined by fitting a Gaussian mixture model (GMM) to the data at baseline.

Tau PET acquisition via FTP PET has also been published previously,^16^ with standardized uptake value ratios (SUVRs) referenced to cerebellar gray matter. Our analyses focused on two FTP PET bilateral regions of interest: the entorhinal cortex, a site of early tau deposition, and the inferior temporal (IT) lobe, a site proximal to AD‐related vulnerability.^16^ Fewer follow‐up time points were available for FTP PET (mean [standard deviation (SD)] = 2.5 [0.8] years, range = 1–4 years).

For both tracers, we used non–partial volume corrected (PVC) data due to compounded issues of PVC with longitudinal data.^17^ For sensitivity analyses, we conducted analyses with PVC to ensure consistent findings. For our PVC pipeline, we used the Müller–Gärtner method.^18^

### 2.5 Structural MRI

Three‐dimensional (3D) structural T1‐weighted MRI scans were acquired using a Siemens 3 Tesla Tim Trio. FreeSurfer software (v.6) was used to segment the T1‐weighted images, and cortical thickness regions of interest (ROIs) were then averaged to form a composite. The regions, based on the AD signature published by Jack et al.,^19^ included entorhinal, IT, middle temporal, and fusiform. For volumetric measurements, hippocampal volume and gray matter volume were adjusted with intercranial volume (ICV) using previously published methods.^20^

### 2.6 Cardiovascular risk

We used baseline cardiovascular disease (CVD) risk, represented by the Framingham Heart Study CVD risk score, as an outcome variable of interest. In short, the Framingham Heart Study CVD metric describes the probability of a cardiovascular event within 10 years, with higher scores representing a greater risk.^21^ The score involves a weighted sum of the following baseline factors: age, sex, antihypertensive treatment, systolic blood pressure, body mass index, history of diabetes, and current cigarette smoking status.

### 2.7 Optimization of longitudinal cognition

**FIGURE 2:**
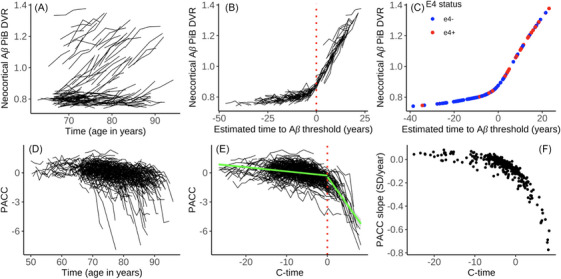
Development of time‐to‐Aβ+ (A–C) and c‐time (D–F). Raw data spaghetti plots of Aβ PET and PACC‐5 trajectories by age over time (B+E); development of time‐to‐Aβ and c‐time metrics with red dashed lines indicating the anchor point. For time‐to‐Aβ+, the anchor point is reaching the Aβ+ threshold and for c‐time, it is at the cognitive inflection point. Finally, the association between each time metric and either baseline neocortical Aβ PET burden or PACC‐5 slope (C+F). Aβ, amyloid beta; c‐time, cognitive time; DVR, distribution volume ratio; PACC‐5, Preclinical Alzheimer Cognitive Composite‐5; PET, positron emission tomography; PiB, Pittsburgh compound B

Each of the following optimization metrics was calculated using publicly available code from the published supplementary materials.^5^ We calculated two metrics to represent individual distances from an anchor point based on cognition and Aβ, respectively (see Figure 2). We developed a c‐time estimate using longitudinal trajectories of the PACC‐5 to distill these data into a single time‐shifted metric. We first subtracted each individual's baseline PACC‐5 score from their subsequent cognitive visit so that all individuals started from a zero point. We then developed a group‐level curve to which all the PACC‐5 trajectories would be optimized. To find the ideal group‐level curve, we explored a series of options, with the most successful curve involving a mixture of linear, quadratic, and exponential components. This model minimized the deviation between the group fit and PACC‐5 trajectories and served as a starting point for the overall trajectory. A subset of individual trajectories was then fit to this curve by optimizing their position using a non‐linear least‐squares fit. The group function was recalculated based on the new trajectory positions, and this process was iterated until the curve parameters converged, minimizing the residual error between the fitted and observed values. All trajectories were fit to this group‐level curve using this optimization process. C‐time values were extracted from final fit positions for each individual, and these values were used for further analyses. To define a meaningful zero for c‐time, we ran iterative piece‐wise linear mixed effects models to determine a “hinge point” at which cognitive slopes prior to a threshold (defined every 0.1 unit along c‐time) were significantly different from cognitive slopes post‐threshold (see supporting information). This threshold was then subtracted from all c‐time numbers to define a new zero that was “anchored” to this threshold. A larger, positive number of the c‐time metric indicates a steeper cognitive decline trajectory, while a smaller, negative number denotes greater stability or even a practice effect.

### 2.8 Optimization of longitudinal Aβ

Time to amyloid positivity (time‐to‐Aβ+) was calculated using a previously published SILA algorithm with the anchor point indicating the Aβ+ threshold.^5^ In brief, the SILA method (1) discretely samples DVR versus age to determine a relationship between the rate of DVR accumulation and the DVR itself, (2) uses numerical integration to find a DVR versus Aβ+ duration curve, with the zero point referring to the threshold of Aβ+, and (3) time‐to‐Aβ+ is estimated by solving this curve for an individual's observed DVR and subtracting the time‐to‐Aβ+ from their age at the observation.

### 2.9 Statistical analysis

C‐time and time‐to‐Aβ+ were calculated in MATLAB (version R2020b), and all other statistical analyses were conducted in R (version 4.2.2). Scripts for c‐time can be found in the following GitHub repository: https://github.com/dtownsend2022/c‐time.

C‐time was validated using a survival analysis using a Cox proportional hazards model to examine the association between c‐time and progression to cognitive impairment or dementia. This method was further validated by applying SILA to the PACC‐5. All analyses were run with PACC replacing c‐time, and PiB PET replacing time‐to‐Aβ+.

We ran a series of non‐parametric Spearman correlations, Kruskal–Wallis associations, or linear regressions adjusting for age to determine the relationship between c‐time and time‐to‐Aβ+ and the following variables of interest: age, sex, apolipoprotein E (*APOE*) ε4 carrier status, education, baseline Aβ, baseline ICV‐adjusted gray matter and hippocampal volume, baseline cortical thickness, and first timepoint of entorhinal and IT tau (SUVR) and baseline cardiovascular risk. We also examined associations of c‐time and time‐to‐Aβ+ with longitudinal tau, brain atrophy (gray matter and hippocampal volume), and cortical thinning using linear mixed effects models, adjusting for age and sex.

We conducted an exploratory mediation analysis to examine the extent to which tau PET signal and hippocampal volume, each in isolation, mediated the association between time‐to‐Aβ+ and c‐time after adjusting for age. Given the multifactorial nature of disease progression, we then extended these models using path analyses model associations between tau PET, cortical thickness, cardiovascular disease risk, time‐to‐Aβ+, and c‐time, while adjusting for age, sex, and *APOE* ε4 carrier status.

Finally, we constructed brain surface maps to show how c‐time and time‐to‐Aβ+ are associated with vertex‐wise FTP PET at the final time point (model 1; cross‐sectional) and vertex‐wise cortical thickness at the final time point (model 2; cross‐sectional). Each of these exploratory vertex‐wise maps adjusted for age, sex, and *APOE* ε4 status, and was thresholded using false discovery rate correction.

## 3 RESULTS

### 3.1 Validating c‐time

The Cox proportional hazards model revealed that c‐time was significantly associated with progression to cognitive impairment/dementia, with a hazard ratio (HR) of 2.04 (± 0.08; *p* < 0.0001). SILA was unable to accommodate all individuals in the dataset, especially those who did not experience the decline. Additionally, replacing c‐time and time‐to‐Aβ+ with PACC and PiB PET, respectively, resulted in qualitatively similar results.

### 3.2 Baseline associations among c‐time, time‐to‐Aβ+, demographics, and AD biomarkers

**FIGURE 3:**
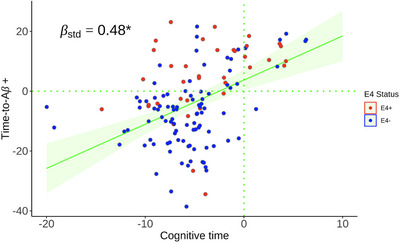
The correlation between c‐time (*x*) and time‐to‐Aβ+ (y; ρ = 0.38, *p *< 0.001) at baseline. Red indicates individuals who are *APOE* ε4+, while those in blue are *APOE* ε4–. Aβ, amyloid beta *APOE*, apolipoprotein E; c‐time, cognitive time

C‐time and time‐to‐Aβ+ both appear symmetrical at baseline (skewness = 0.006; −0.067), but c‐time is leptokurtic (kurtosis = 4.33; Jarque–Bera, *p* < 0.05), while time‐to‐Aβ+ was normal (kurtosis = 2.49; Jarque–Bera, *p* > 0.05). The mean of both distributions were in the negative (mean [SD] = –11.5 [4.4]) and time‐to‐Aβ+ (mean [SD] = −10.2 [13.5]) zero‐point (Figure S1 in supporting information), indicating that most individuals were estimated to be prior to cognitive decline or Aβ abnormality thresholds. C‐time and time‐to‐Aβ+ were moderately, but significantly, correlated (*r* = 0.38, *p* < 0.001; *r* ^2^ = 0.14), with a higher (worse) c‐time associated with a higher (worse) time‐to‐Aβ+ (β_std _= 0.48, *p* < 0.001, when adjusting for age; Figure 3). For both c‐time and time‐to‐Aβ+ indices, higher entorhinal and IT tau PET signal was significantly correlated with higher (worse) scores, with c‐time demonstrating slightly stronger associations (see Figure S2 and Table S1 in supporting information). For c‐time, older age was significantly associated with higher (worse) c‐time, as well as lower gray matter and hippocampal volume, and greater cortical thinning (Table S1 and Figure S3 in supporting information). For time‐to‐Aβ+, *APOE* ε4 carriers exhibited significantly higher (worse) time‐to‐Aβ+ than non‐carriers (*p* < 0.0001), and higher time‐to‐Aβ+ was associated with greater cortical thinning (Table S1 and Figure S3). There were no significant sex differences in either c‐time or time‐to‐Aβ+.

### 3.3 Association of c‐time and time‐to‐Aβ+ with longitudinal change in AD biomarkers

**FIGURE 4:**
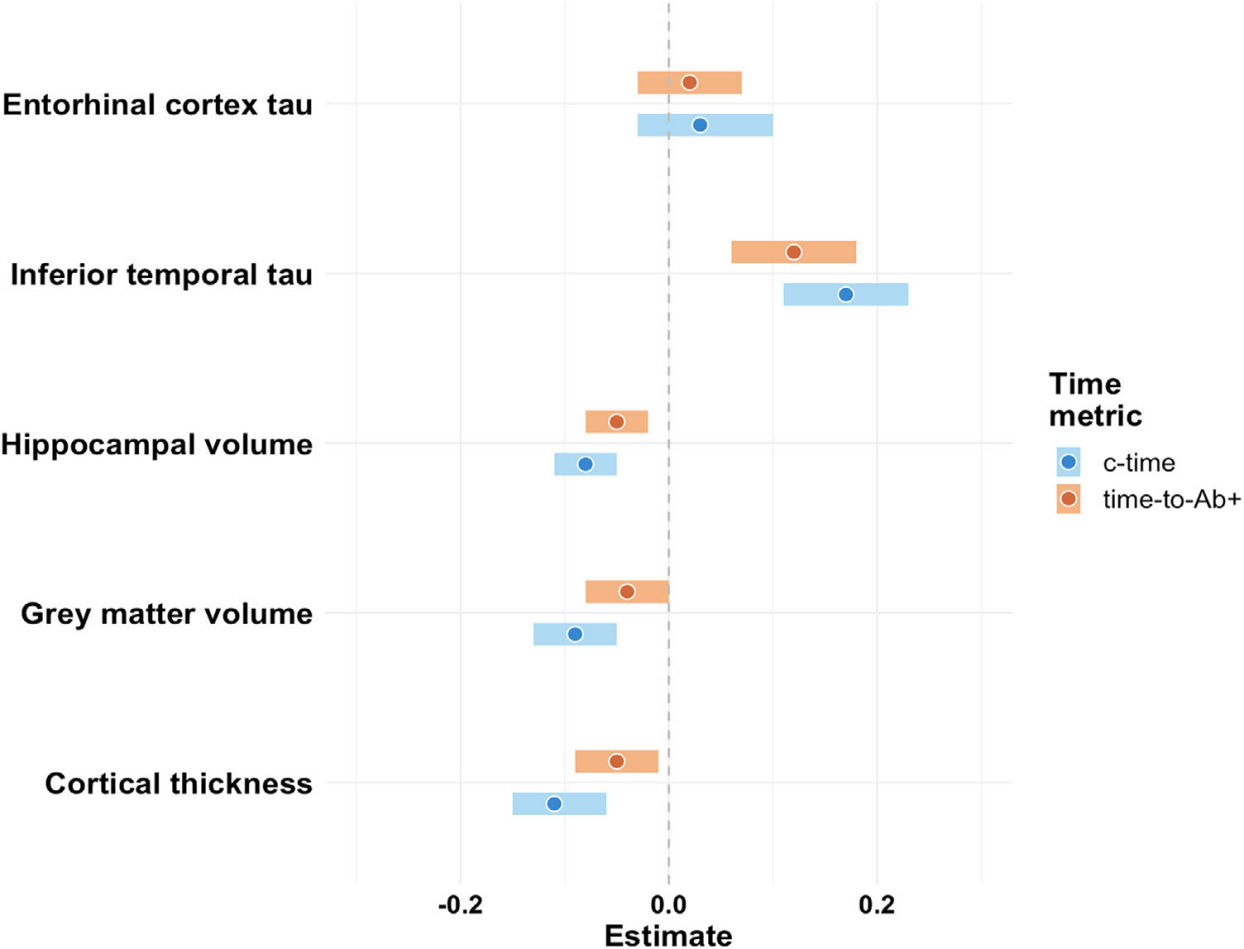
Estimates (*x* axis) resulting from linear mixed effects models showing the effect of c‐time (blue) and time‐to‐Aβ+ (orange) on longitudinal changes in entorhinal tau PET, inferior temporal tau PET, hippocampal volume, gray matter volume, and cortical thickness (*y* axis). Aβ, amyloid beta; CI, confidence interval; c‐time, cognitive time; PET, positron emission tomography

Using linear mixed effects models, we found that higher c‐time, and time‐to‐Aβ+ to a lesser degree, was associated with faster IT tau PET accumulation, hippocampal atrophy, gray matter atrophy, and cortical thinning, after adjusting for age and sex (see Figure 4). Neither metric was associated with changes in entorhinal tau PET.

### 3.4 Mediation and path analysis

**FIGURE 5:**
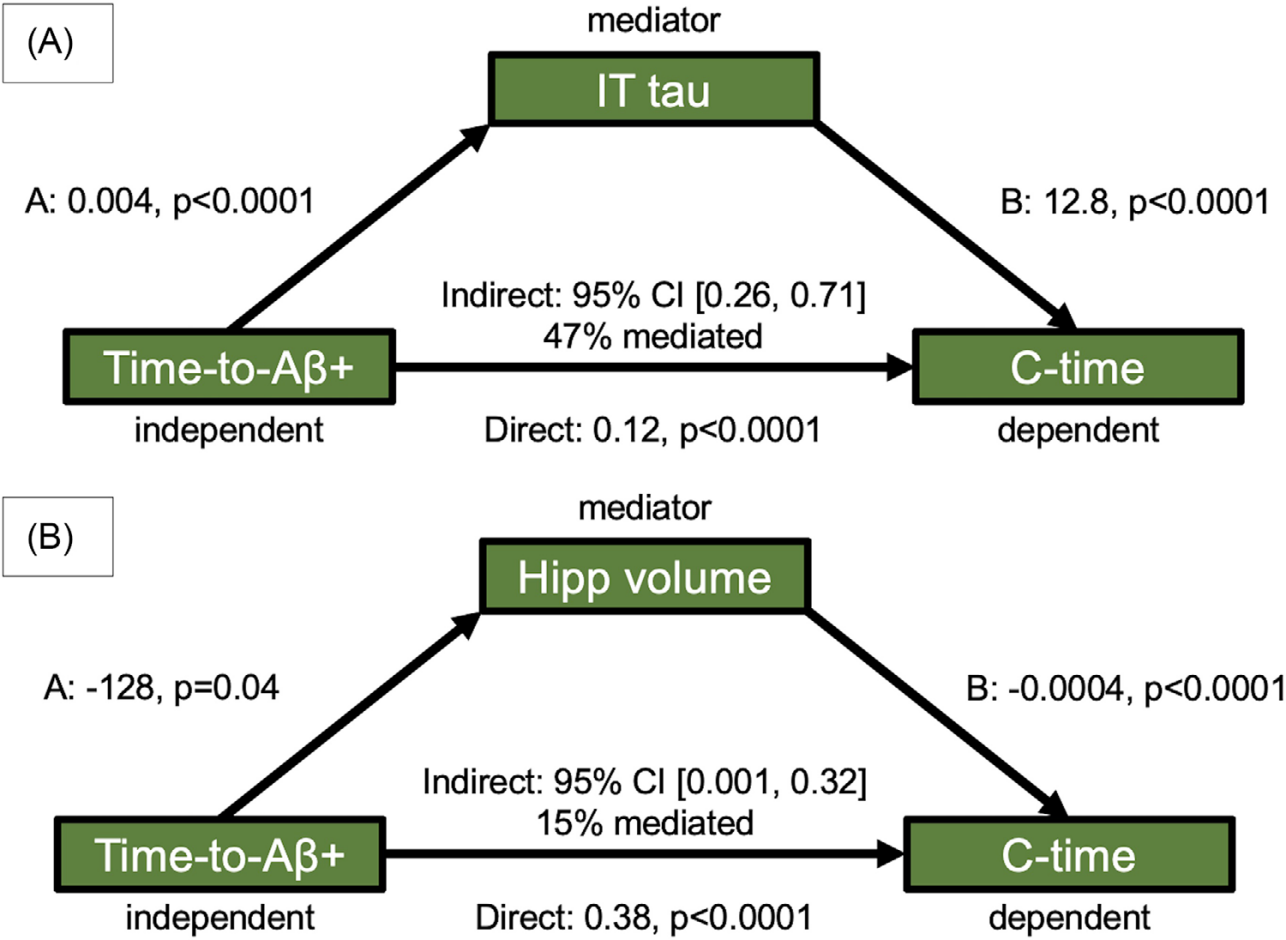
A, Mediation analysis with IT tau acting as the mediator between time‐to‐Aβ+ and c‐time. The total effect of time‐to‐Aβ+ on c‐time is significant (*p *< 0.0001), as well as the effect of time‐to‐Aβ+ on IT tau (*p *< 0.0001), as well as IT tau on c‐time (*p *< 0.0001). This indicates an incomplete mediation (47% mediated), as time‐to‐Aβ+ continues to have an effect on c‐time when controlling for IT tau. B, Hippocampal volume also only partially mediates (15% mediated) the relationship between time‐to‐Aβ+ and c‐time, with the total effect of time‐to‐Aβ+ on c‐time (*p *< 0.0001), time‐to‐Aβ+ on hippocampal volume (*p *= 0.04), and hippocampal volume on c‐time (*p *< 0.0001) being significant. Aβ, amyloid beta; c‐time, cognitive time; IT, inferior temporal

**FIGURE 6:**
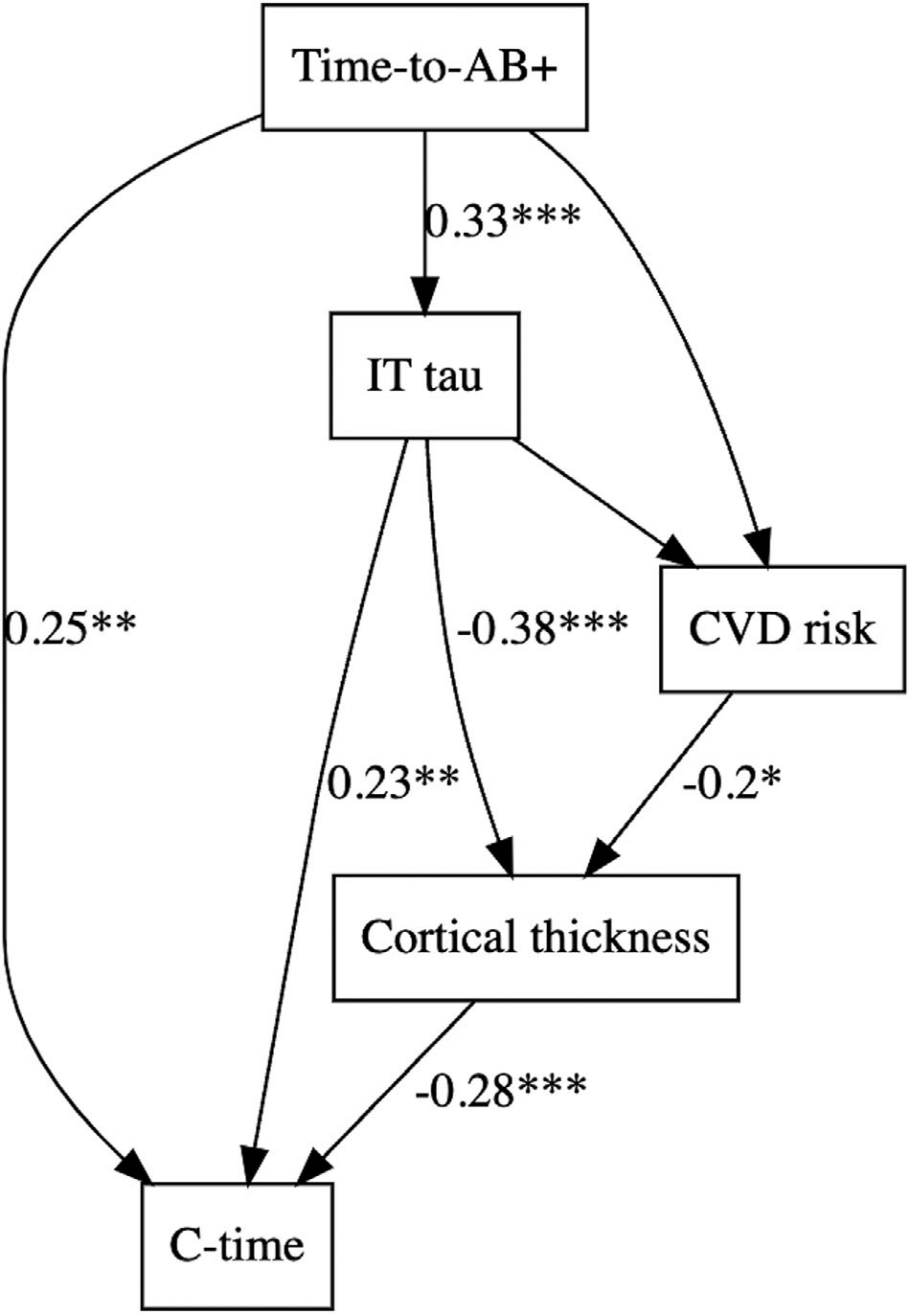
Path diagram showing the standardized regression paths between time‐to‐Aβ+, inferior temporal tau PET, cardiovascular disease risk, cortical thickness, and c‐time. Significant paths are labeled with *p* values and have been adjusted for age, sex, and *APOE* ε4 carrier status. Aβ, amyloid beta; *APOE*, apolipoprotein E; c‐time, cognitive time; CVD, cardiovascular disease; PET, positron emission tomography

Two separate mediation analyses showed a partial mediation effect of IT tau PET (47% mediation) and hippocampal volume (15% mediation), respectively, on the association between time‐to‐Aβ+ and c‐time, after adjusting for age (Figure 5). More expansive path analyses integrated IT tau PET, hippocampal volume, cortical thickness, and cardiovascular risk as mediators between time‐to‐Aβ+ and c‐time after adjusting for age, sex, and *APOE* ε4 carrier status (Figure 6). These analyses revealed a direct effect between time‐to‐Aβ+ and c‐time, as well as indirect effects via IT tau PET and cortical thickness. Additionally, CVD risk showed no direct association with time‐to‐Aβ+ or c‐time but showed a negative indirect association on c‐time via cortical thickness.

### 3.5 Brain surface maps versus cognitive and amyloid time

Exploratory brain surface maps revealed that the distribution and magnitude of tau PET signal across the brain were more closely associated with c‐time than time‐to‐Aβ+ (see Figure S4 in supporting information). For c‐time, the strongest associations were localized to the medial and lateral temporal cortices, and to a lesser extent, the inferior parietal and lateral occipital regions. For cortical thinning, c‐time showed weak associations in the medial and IT regions. No significant cortical thinning associations were found with time‐to‐Aβ+ (see Figure S4).

## 4 DISCUSSION

In this study of longitudinal and non‐linear cognitive and biomarker trajectories in the HABS, we present a single continuum of cognition, termed c‐time, and examine its association with a previously published time‐to‐Aβ+.^5^ Our c‐time method distills longitudinal cognitive data from multiple individuals into a single continuous number that represents an individual's distance from a group‐level “hinge point” of cognitive decline. The benefit of optimizing longitudinal cognitive data is to reduce complex, individual trajectories across multiple time points into a single value per participant, providing their relative position along a continuum within the sample. An advantageous byproduct of this approach is that it effectively removes some statistical complexities associated with modeling non‐linear cognitive change through methods such as splines. Rather than discarding important heterogeneity in the PACC trajectories, c‐time reframes it: individuals with steeper decline, for example, are positioned later along the curve, while those with stable or improving performance are positioned earlier. In this way, c‐time complements, rather than replaces, traditional approaches that examine variability in slopes or non‐linear change. While longitudinal trajectories of amyloid burden and PACC indeed offer important information, our goal with c‐time was not to replace these raw trajectories, but to develop a single, optimized, time‐based metric. By substituting PACC and PiB PET measures in place of c‐time and time‐to‐Aβ+ in our models, we yielded qualitatively similar results. Like previously published frameworks,^10^ our goal was not to demonstrate superior predictive performance over raw trajectories, but to provide a statistically and clinically interpretable disease time scale despite the lack of definitive diagnostic endpoints in preclinical AD.

We found c‐time to be more closely associated with demographics, cross‐sectional and longitudinal changes in tau PET, brain atrophy, and cortical thickness than time‐to‐Aβ+. These stronger associations between c‐time and downstream biomarkers (e.g., tau, atrophy) likely reflect the multifactorial nature of cognitive decline, which encompasses, but is not limited to, the Aβ cascade. C‐time may thus capture a more integrative signal of disease progression than time‐to‐Aβ+ alone. While c‐time and time‐to‐Aβ+ were moderately associated, we recapitulated many indirect pathways between the two metrics, via tau PET, cortical thinning, and brain atrophy, supporting previous literature,^22^, ^23^, ^24^ and further validating the position of c‐time within the established AD cascade. Further, CVD risk provided an indirect association with c‐time via cortical thickness that was entirely independent of time‐to‐Aβ+. Together, these results provide a strong validation for this methodology within a preclinical AD cohort, and an alternative way of modeling a cognitive continuum in a sample with no definitive diagnostic endpoint.

Many studies have developed a time metric from biomarkers or cognition to better understand disease progression for AD.^5^, ^7^, ^8^, ^9^, ^25^, ^26^, ^27^, ^28^, ^29^, ^30^ In the past, these models have relied entirely on a time‐to‐event, such as meeting a threshold or diagnosis. Observational studies of predominantly clinically normal older adults cannot rely on these endpoints, given that rates of progression to dementia are so few. Our approach capitalizes on the length of cognitive follow‐up and the group‐level curvature to model a continuum.

Our work validates previous studies of disease progression models,^4^, ^5^, ^7^, ^9^, ^27^, ^28^ which coalesce on the strategy of aligning longitudinal data along a global curve and allotting each individual to a shifted placement on that continuum relative to the rest of the sample. The development of c‐time is effective in that it has fewer statistical assumptions than many models and aligns well with time‐to‐Aβ+ using the SILA method.^5^ Unlike time‐to‐Aβ+, however, c‐time was more closely associated with biomarkers and atrophy both at baseline and longitudinally and was effective in representing all individuals along the cognitive spectrum. This supports the ample evidence showing that cognitive decline is proximal to tauopathy and neurodegeneration.^22^, ^28^

This method for estimating an individual's distance (time) to a certain event is not without limitations. First and foremost, the optimization method is sensitive to the number of observations available for each individual as well as the mixture proportion of the sample (i.e., frequency of females, *APOE* ε4 carriers, those with abnormal Aβ burden, etc.). There is a lack of diversity in this sample, with the majority of individuals self‐reporting as non‐Hispanic White, demonstrating high levels of education and low cardiovascular risk. Further, practice effects in the cognitive trajectories make the curvature estimation more challenging. C‐time is also relative to the rest of the group, meaning that it is not anchored to an external outcome like diagnosis. Further comparisons to other cohorts might allow for harmonization and elimination of these issues. Finally, we implemented different optimization methods for cognition and Aβ. We did not implement the SILA approach for c‐time because it was unable to adequately model individuals with consistently high cognitive performance or practice effects over time. Specifically, these participants could not be incorporated into the shared group trajectory in SILA, leading to their exclusion and reduced generalizability across the full cognitive spectrum. We did, however, retain SILA for estimating time‐to‐Aβ+ because its design—anchoring to a biomarker threshold and assuming relatively uniform progression—is well suited to the sigmoidal trajectory of Aβ accumulation. Moreover, SILA has been validated across multiple large cohorts and has become a well‐established method in the field.^5^, ^31^

C‐time provides a new method for understanding an individual's placement on a cognitive continuum and, along with time‐to‐Aβ+, can give us unique, important information about the progression of preclinical AD. The ability to predict an individual's “preclinical timeline” will be effective for more robustly modeling early changes in disease while avoiding non‐linear complexities when modeling the data. This method can be applied to other longitudinal cognitive studies by giving age‐independent and individual‐specific information about the time to any disease‐related event, which is key for pre‐clinical intervention.

## CONFLICT OF INTEREST STATEMENT

All authors report no disclosures relevant to this manuscript. Author disclosures are available in the supporting information.

## CONSENT STATEMENT

All subjects provided written informed consent.

## Supporting information



Supporting Information

Supporting Information

## Data Availability

HABS data are publicly available for request at https://habs.mgh.harvard.edu/researchers/request‐data/
