# Supplementary material for: Estimating the preclinical Alzheimer's disease course with multimodal data
Source: Alzheimers Dement. 2025 Sep 3;21(9):e70658. doi: 10.1002/alz.70658 (PMC12405800; doi:10.1002/alz.70658)
Supplement: Supplementary file 2 — Supporting Information [file ALZ-21-e70658-s002.docx]

**Supplemental**


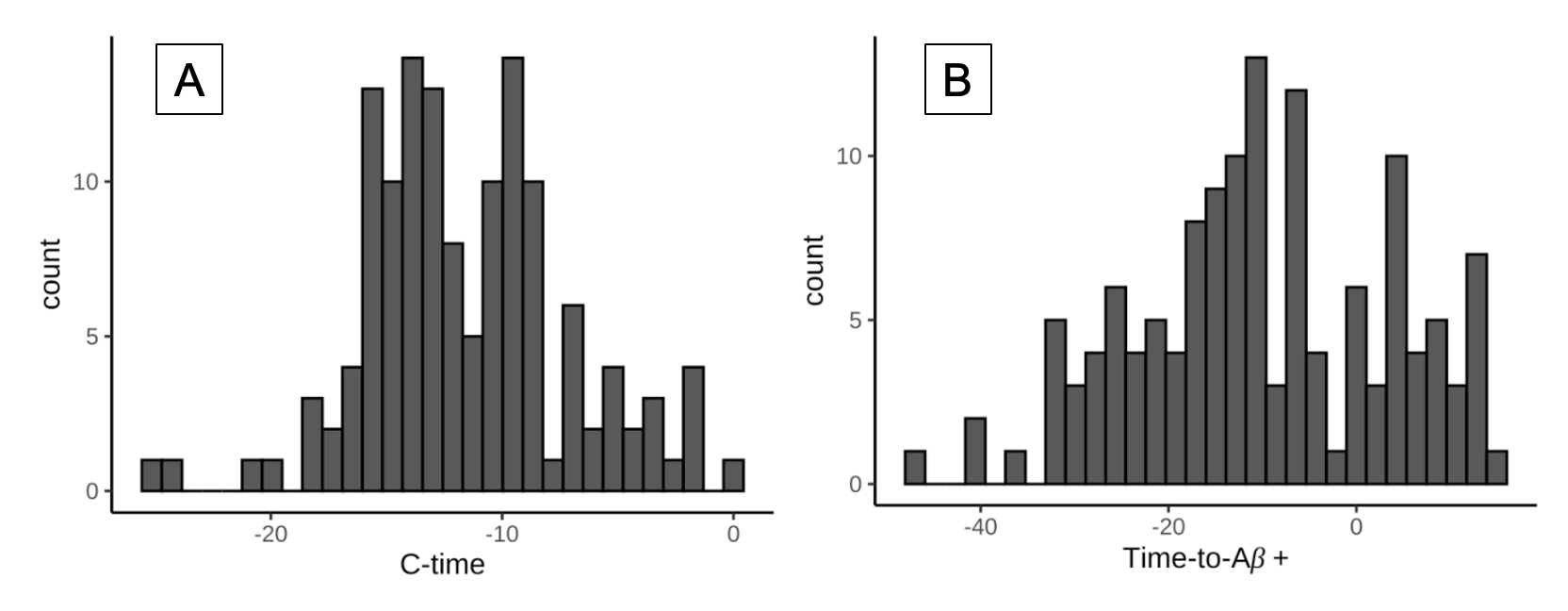


**Supplementary Figure 1.** Histograms of c-time (A) and time-to-Aβ+ (B) for participants at baseline.


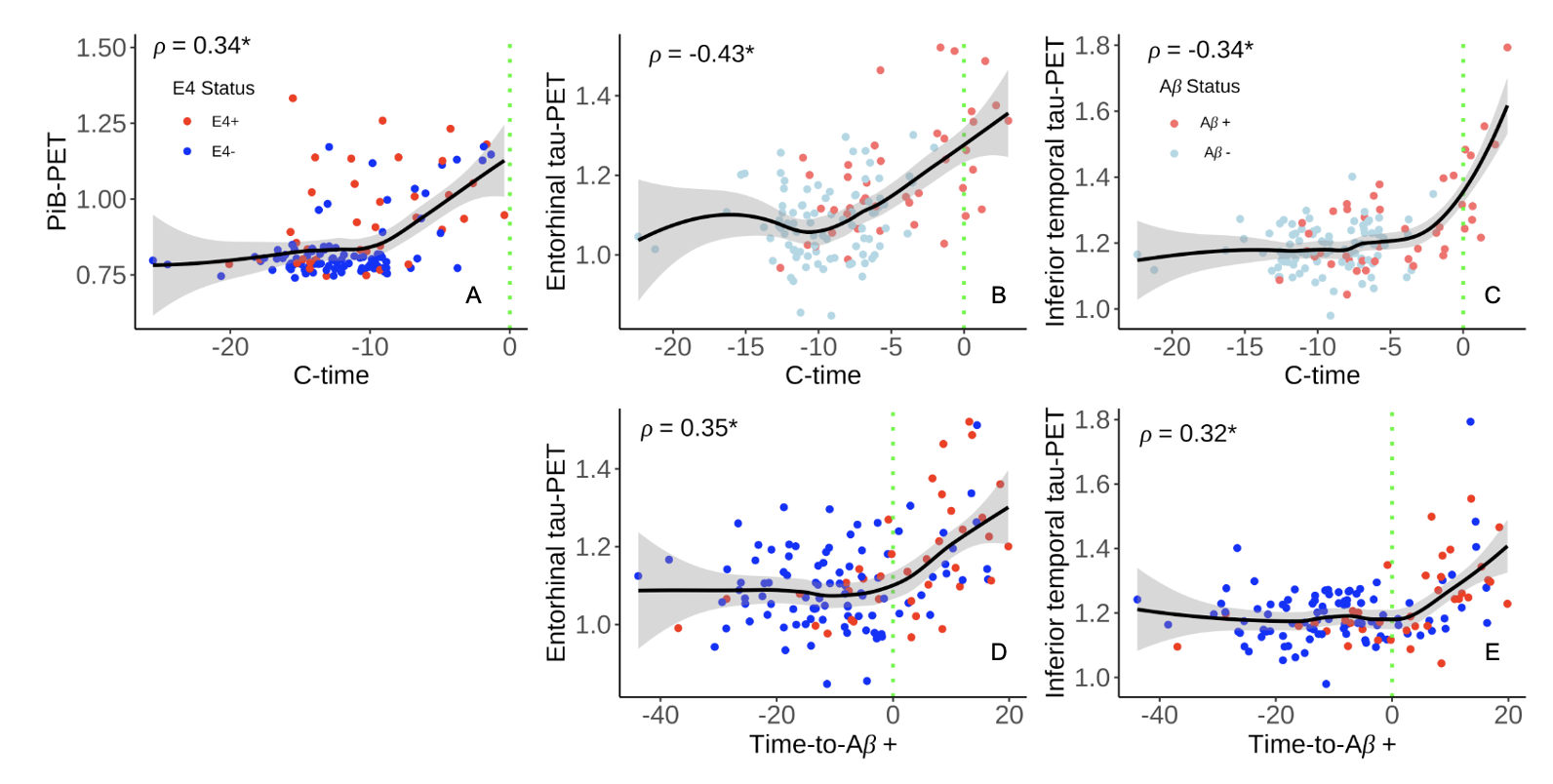


**Supplementary Figure 2.** Baseline amyloid-PET (A), and entorhinal (B) and inferior temporal (C) tau-PET over c-time and baseline entorhinal cortex (D) and inferior temporal tau-PET (E) over time-to-Aβ+, with ρ representing the Spearman correlation. Red points indicate APOEε4 carriers, with blue as non-carriers. Light red represents those who are amyloid positive (threshold of 0.87 PiB-PET) and light blue representing those who are amyloid negative. Green dashed line represents c-time and time-to-Aβ+ thresholds.

**Supplementary Table 1**. Associations (Spearman’s correlation for age and linear regressions adjusting for age for the remaining biomarkers) between demographics and biomarkers and c-time/time-to-Aβ+. Bold numbers indicate significant correlations.

| **Demographic/biomarker** | **C-time** | **Time-to-Aβ+** |
| --- | --- | --- |
| **Age** | **ρ = 0.48 (*p*<0.0001)** | ρ = 0.17 (*p*=0.06) |
| **EC FTP SUVR** | **β_std_ = 0.51 (*p*<0.001)** | **β_std_ = 0.38 (*p*<0.001)** |
| **IT FTP SUVR** | **β_std_ = 0.5 (*p*<0.001)** | **β_std_ = 0.36 (*p*<0.001)** |
| **Hippocampal volume adjusted** | **β_std_ = -0.23 (*p*=0.004)** | β_std_ = -0.02 (*p*=0.80) |
| **Grey matter volume adjusted** | **β_std_ = -0.27 (*p*=0.002)** | β_std_ = -0.03 (*p*=0.75) |
| **Cortical thickness** | **β_std_ = -0.36 (*p*<0.001)** | **β_std_ = -0.19 (*p*=0.03)** |
| **Cardiovascular disease risk** | β_std_ = 0.16 (*p*=0.06) | β_std_ = 0.08 (*p*=0.29) |


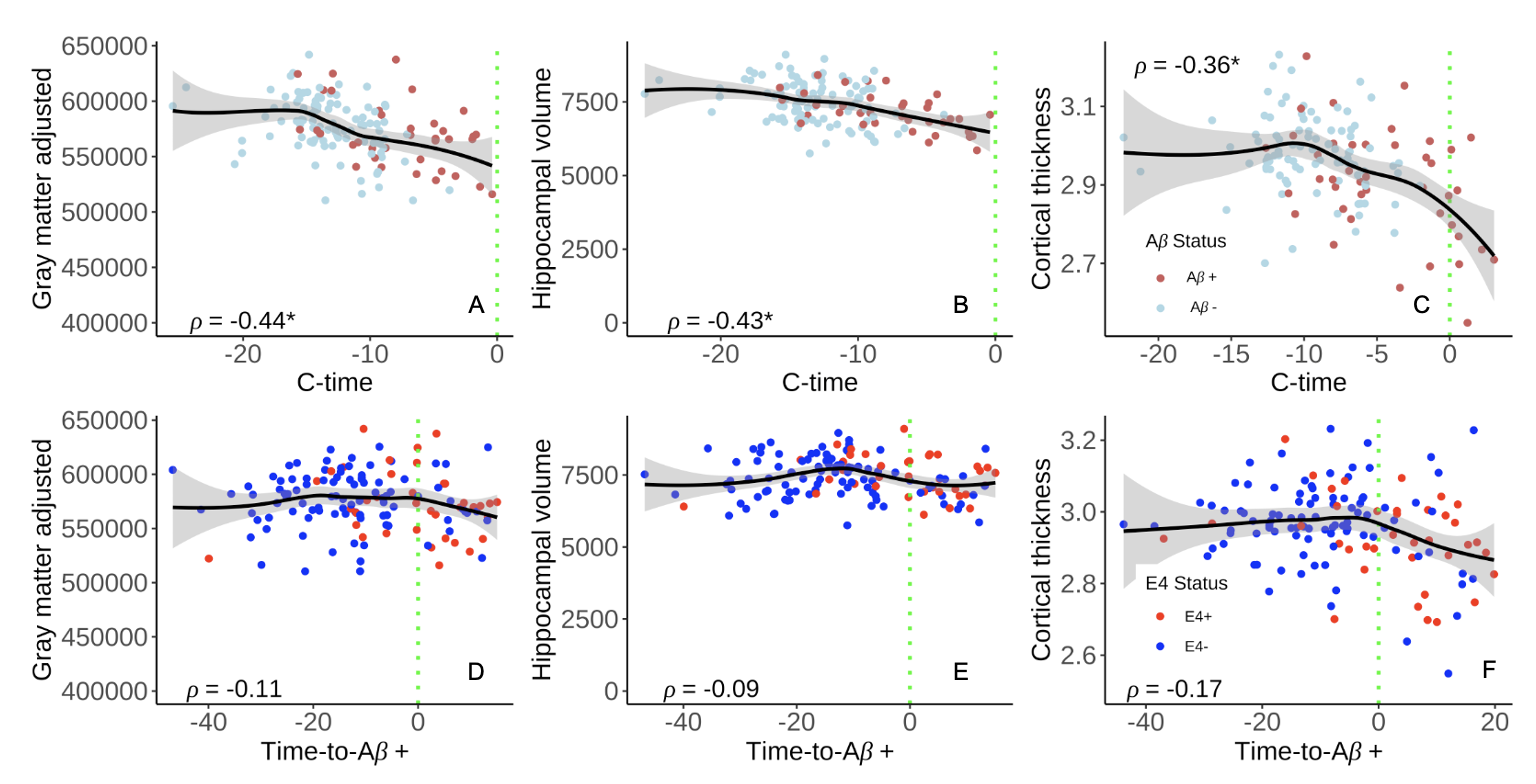


**Supplementary Figure 3.** Baseline gray matter volume (A, D), hippocampal volume (B, E), and cortical thickness (C, F) as a function of c-time and time-to-Aβ+, respectively, with ρ representing the Spearman correlation. Light red points represent those who are amyloid positive (0.87 PiB-PET threshold), while those who are light blue are negative. Red points indicate APOEε4 carriers, with blue as non-carriers. Green dashed line represents c-time and time-to-Aβ+thresholds.

**
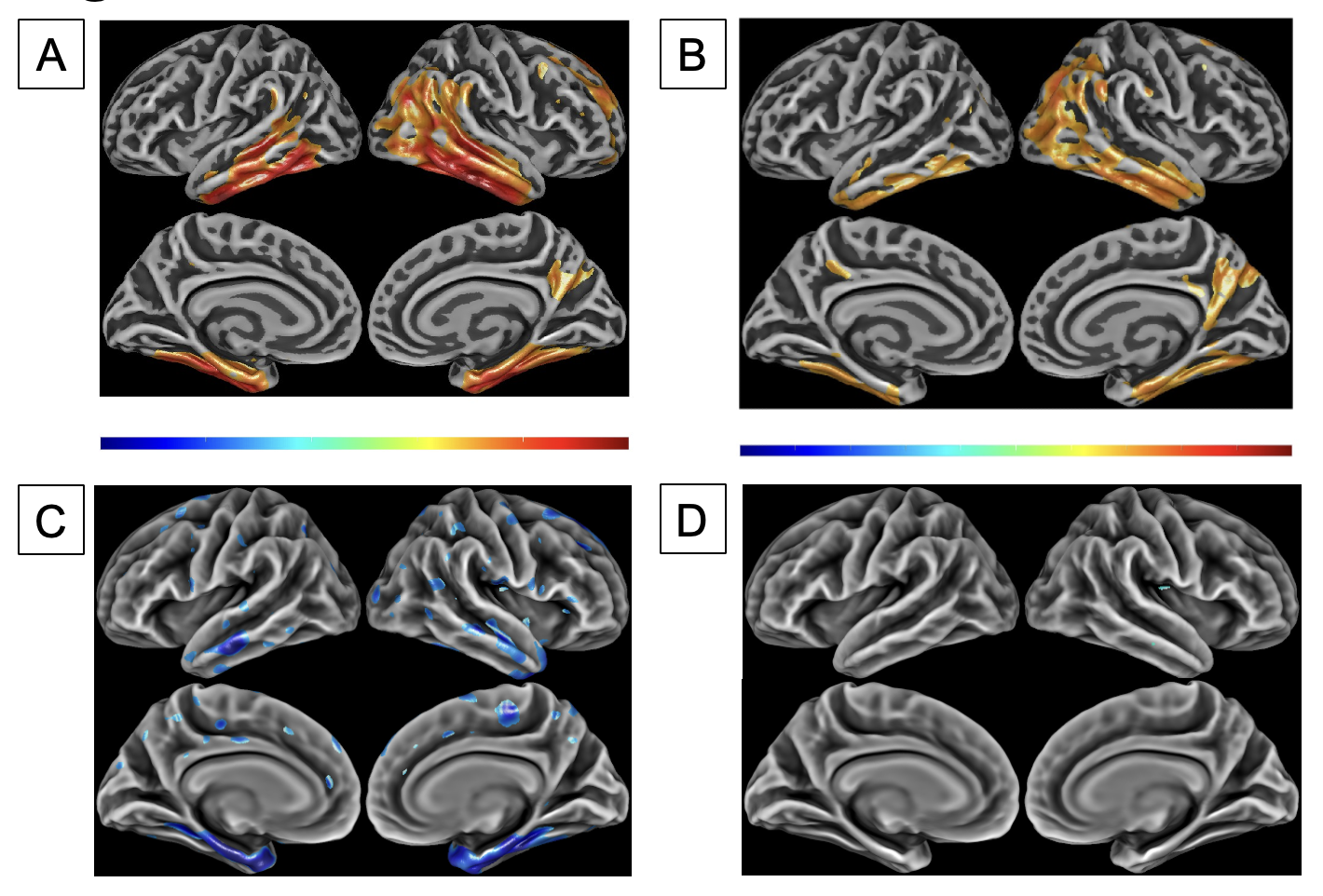
**

**Supplementary Figure 4.** Brain surface maps showing the strength and distribution of the association of tau-PET with c-time (A) and time-to-Aβ+ (B) and cortical thinning associated with c-time (C) and time-to-Aβ+ (D). Warmer colors indicate a stronger association.
